# The morphology of human rod ERGs obtained by silent substitution stimulation

**DOI:** 10.1007/s10633-017-9571-4

**Published:** 2017-01-13

**Authors:** J. Maguire, N. R. A. Parry, J. Kremers, I. J. Murray, D. McKeefry

**Affiliations:** 1 Bradford School of Optometry and Vision Sciences, University of Bradford, Bradford, W. Yorkshire BD7 1DP UK; 2 Vision Science Centre, Manchester Royal Eye Hospital, Central Manchester University Hospitals NHS Foundation Trust, Manchester Academic Health Science Centre, Manchester, UK; 3 Faculty of Biology, Medicine and Health, University of Manchester, Manchester, UK; 4 Department of Ophthalmology, University Hospital Erlangen, Erlangen, Germany

**Keywords:** Electroretinograms, Rod photoreceptors, Silent substitution

## Abstract

**Purpose:**

To record transient ERGs from the light-adapted human retina using silent substitution stimuli which selectively reflect the activity of rod photoreceptors. We aim to describe the morphology of these waveforms and examine how they are affected by the use of less selective stimuli and by retinal pathology.

**Methods:**

Rod-isolating stimuli with square-wave temporal profiles (250/250 ms onset/offset) were presented using a 4 primary LED ganzfeld stimulator. *Experiment 1*: ERGs were recorded using a rod-isolating stimulus (63 ph Td, rod contrast, *C*_rod_ = 0.25) from a group (*n* = 20) of normal trichromatic observers. *Experiment 2*: Rod ERGs were recorded from a group (*n* = 5) using a rod-isolating stimulus (*C*_rod_ = 0.25) which varied in retinal illuminance from 40 to 10,000 ph Td. *Experiment 3*: ERGs were elicited using 2 kinds of non-isolating stimuli; (1) broadband and (2) rod-isolating stimuli which contained varying degrees of L- and M-cone excitation. *Experiment 4:* Rod ERGs were recorded from two patient groups with rod monochromacy (*n* = 3) and CSNB (type 1; *n* = 2).

**Results:**

The rod-isolated ERGs elicited from normal subjects had a waveform with a positive onset component followed by a negative offset. Response amplitude was maximal at retinal illuminances <100 ph Td and was virtually abolished at 400 ph Td. The use of non-selective stimuli altered the ERG waveform eliciting more photopic-like ERG responses. Rod ERGs recorded from rod monochromats had similar features to those recorded from normal trichromats, in contrast to those recorded from participants with CSNB which had an electronegative appearance.

**Conclusions:**

Our results demonstrate that ERGs elicited by silent substitution stimuli can selectively reflect the operation of rod photoreceptors in the normal, light-adapted human retina.

## Introduction

The human electroretinogram (ERG), when elicited by a diffuse flash of light, constitutes a global electrical response from the retina which reflects the neural activity of a number of different retinal cell populations. However, with careful choice of the temporal, chromatic and luminance characteristics of the stimulus, it is possible to generate responses that have a greater degree of specificity in terms of the retinal cell populations from which they originate [1]. The isolation and selective stimulation of rod photoreceptor activity form an important part of clinical electrodiagnostic assessment routines. There is a variety of congenital and acquired visual pathologies that can differentially affect rod relative to cone function [2–7]. The International Society for Clinical Electrophysiology of Vision (ISCEV) has outlined a detailed set of standards governing all aspects of clinical electroretinography [8] which covers scotopic (and photopic) retinal assessment. However, in recent years, other non-standard test methods have been developed and these have proven to be useful in providing extra information about retinal function. One method that has become popular, following the wider availability of four and five primary LED stimulator systems, is silent substitution [9, 10]. This method provides a means by which ERGs from any one of the retinal photoreceptor populations can, in theory, be isolated from the other photoreceptor classes. In the case of rod isolation, four primary stimulators allow the creation of stimuli which, when modulated in time, produce a constant level of photoisomerisations in the three types of cone photoreceptors, but not in the rods [11, 12]. Thus, cone modulation is effectively kept at zero while the rods are selectively stimulated.

In a previous study, we demonstrated that it is possible to isolate rod-mediated steady-state (8 Hz) ERG responses using the silent substitution method without the need for dark adaptation [13]. We were able to show that ERGs elicited by this technique were selective for rods by the demonstration of a correspondence between temporal frequency and illuminance response characteristics and previously reported psychophysical properties of rod-mediated vision. In this study, we have used the same silent substitution technique to generate transient ERGs using stimuli with square-wave temporal profiles. This approach facilitates examination of rod-mediated responses in the time domain and enables characterisation of the morphology of the ERG waveform and its constituent components. The primary aim of this study was to describe the basic morphological features of the ERG associated with rod function in the normal trichromatic retina generated by silent substitution stimuli. In addition, we also wanted to explore how the rod ERG waveform morphology is affected by the use of less selective stimuli that modulate cone as well as rod photoreceptors. To this end, we compared rod-isolated ERGs, elicited by silent substitution, with responses obtained using non-selective broadband ‘white’ stimuli and stimuli to which we intentionally introduced varying degrees of cone modulation. Such stimulus manipulations allow us to identify key changes in the ERG waveform that might be attributable to the intrusion of cone activity. We also wanted to explore interactions between rod and cone responses using stimuli of varying intensities. Of particular interest is the way the rod ERG waveform is influenced by the use of stimuli that span the mesopic illumination range. This range is important as it marks the main transition between rod- and cone-mediated visual function, and it would be useful to ascertain whether the rod ERG reflects this transition in the human retina, as has been previously demonstrated in the mouse [14].

As well as examining rod-mediated ERGs from the normal trichromatic human retina, we also wanted to assess responses from individuals with specific retinal pathologies. In the context of this study, individuals with rod monochromacy constitute an important control group. Such individuals lack significant cone function and effectively only possess functioning rod photoreceptors. Thus, rod ERGs from these individuals can be compared to those responses obtained from normal trichromats (who still have functioning L-, M- and S-cones). If our silent substitution stimuli and recording conditions do effectively isolate rod function, then we would expect a high degree of correspondence between the morphological features of ERGs elicited from normal trichromats and those from rod monochromats. To facilitate this comparison, we recorded rod ERGs from subjects who have rod monochromacy caused by CNGB3 gene mutations. Such mutations result in completely or highly impaired cone function which results in abnormal colour vision, reduced visual acuity and nystagmus [15–17]. Conversely, other retinal pathologies, such as the complete form of congenital stationary night blindness (CSNB1), for example, lead to severely compromised rod function but preserved cone function [18]. CSNB1 is associated with ON-bipolar cell dysfunction and leads to a characteristic set of full-field ERG abnormalities including abolished scotopic rod responses, electronegative mixed rod-cone responses and preserved, though abnormal, photopic responses [18–20]. In such cases, we would expect rod responses generated by silent substitution stimuli in participants with CSNB1 to be very different from those obtained from those with normal retinal function. The comparison of rod ERGs generated by silent substitution in participants with normal as well as pathological retinal function is useful as it will help us to gauge the extent to which our methodological approach leads to the effective and selective isolation of rod photoreceptoral function in humans.

## Methods

### Stimuli

**Fig. 1:**
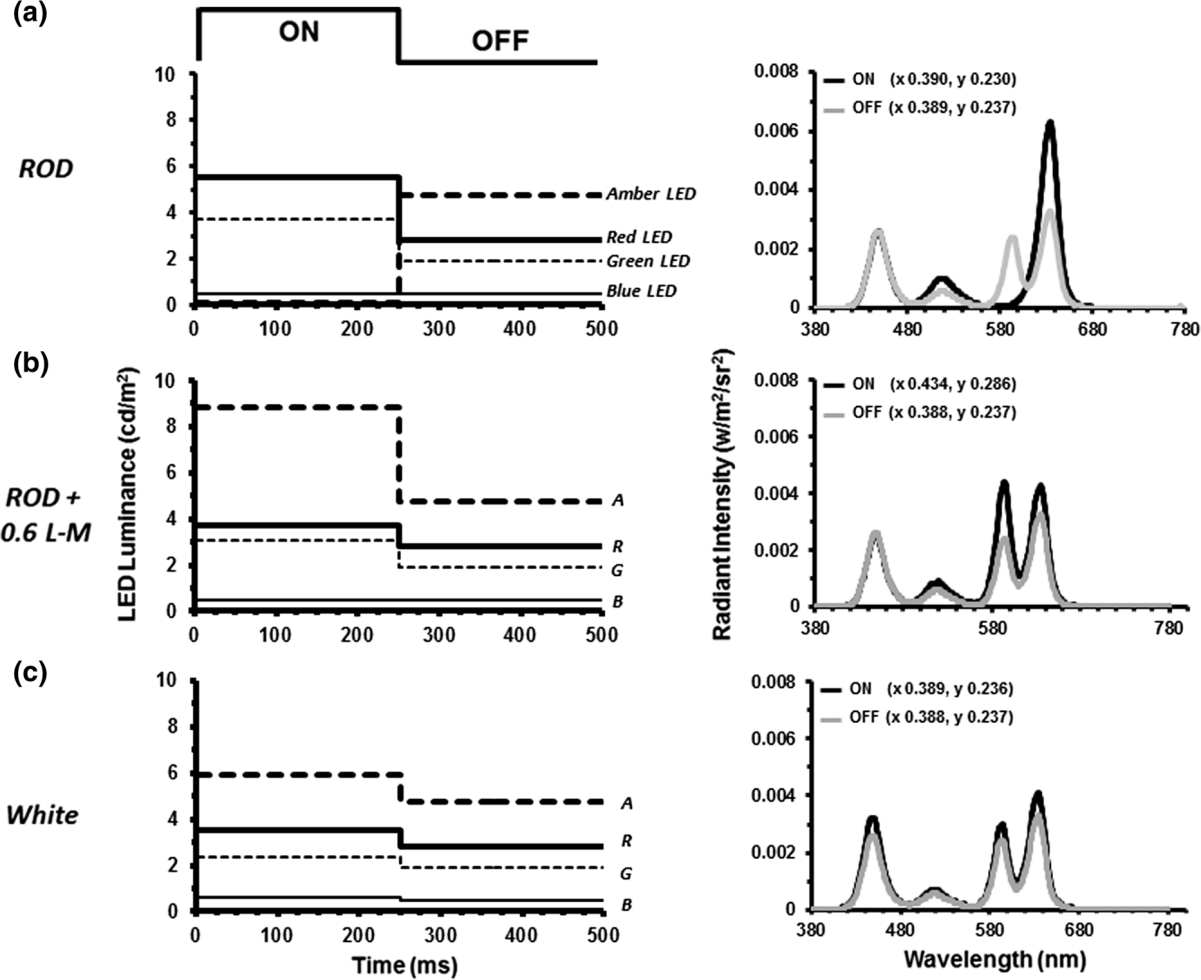
Temporal profiles of the square-wave pulse stimulus used to generate rod ERGs. The plots on the *left* show the luminance variation of the four LED primaries required to generate: **a** the rod-isolating stimulus, **b** the mixed rod and L- and M-cone stimulus (cone modulation = 0.6) and **c** the ‘white’ stimulus. In each case, the initial 0–250 ms is the onset period followed by the offset period (250–500 ms). This sequence was then repeated with the stimuli presented as continuous trains of on–off pulses (256 cycles in total). The graphs on the *right-hand side* show the spectral characteristics of the onset (*black lines*) and offset (*grey lines*) phases of each of the stimuli. Also given are the 1931 CIE (xy) chromaticity co-ordinates for the onset and offset phases of each stimulus

Rod-isolating stimuli were presented using a ColorDome (Diagnosys LLC, Lowell, MA, USA) four primary ganzfeld stimulator with blue (460 nm), green (514 nm), amber (592 nm) and red (632 nm) LEDs. The spectral characteristics, chromaticities and luminances of each class of LED were calibrated using a PR650 spectrophotometer (Photo Research Inc., Chatsworth, CA, USA). In order to create silent substitution stimuli, photoreceptor excitations were calculated by multiplying the emission spectra of the LEDs with cone fundamentals and the V’_λ_ 10° function [22, 23] and integrating over a range of wavelengths (see: Ref. [13] for a fuller description of stimulus generation). The stimuli used in these experiments were triple silent substitutions in which intensity and wavelength combinations were used which produced no change in the net excitation of L-, M- or S-cones, but did produce excitation modulation of rod photoreceptors. Figure 1a illustrates an example of a rod-isolating stimulus. In these experiments, the modulation of rod excitation was kept constant at *C*_rod_ = 0.25 (Michelson contrast) for all stimuli. The retinal illuminance produced by the stimuli was varied between 40 and 10,000 photopic trolands (ph Td). In order to obtain the stimuli with the lowest retinal illuminances (40, 63 ph Td), a 0.9 neutral density filter was placed in front of the stimulator which attenuated the stimuli to the required levels with little or no distortion of the spectral characteristics.

For consistency, we have used photopic as opposed to scotopic Troland units throughout this study rather than change units across the transitional mesopic–photopic illumination range within which the majority of our stimuli lie. Prior to the start of each experimental session, the participants underwent a 5-min adaptation period under ambient room illumination (500 lx). The stimuli were then delivered as continuous trains of pulses (only 1 cycle is shown in the Fig. 1 for clarity) with each waveform constituting the average response to 256 cycles (on–off presentations) of the stimulus.

In addition to the rod-isolating stimuli, we employed two other types of non-isolating stimuli which were designed to elicit excitation of both rod and cone photoreceptors. For one stimulus type, we introduced varying amounts of L- and M-cone modulation, ranging from 0.0–0.6, into our basic rod stimulus (Fig. 1b). The second kind of non-selective stimulus was produced by the modulation in phase of all four LEDs (Fig. 1c). This so-called ‘white’ stimulus (which actually appeared purple to the normal trichromats) produced the same modulation (0.25) across all four classes of photoreceptor.

### ERG recording

ERGs were recorded from the right eye using a silver/nylon corneal fibre electrode (Dept. of Physics and Clinical Engineering, Royal Liverpool University Hospital, UK) referenced to a 9-mm Ag/AgCl electrode (Biosense Medical, Chelmsford, UK) on the outer canthus; a similar electrode was affixed to the forehead to serve as ground. Impedance was maintained below 5 kΩ. Signals were recorded using the Espion E^2^ system (Diagnosys LLC, Lowell, MA, USA) which amplified and filtered (bandwidth = 1 to 300 Hz) the ERGs and digitised them at a rate of 1000 Hz. Retinal responses were acquired over 500 ms epochs with each response being composed of an average of a minimum of 256 epochs. Participants viewed the stimuli monocularly with a dilated pupil (1% Tropicamide) from a distance of 10 cm, and both a chin and head rest were used. Fixation was maintained on a central point which subtended approximately 0.5°.

### Participants

In experiment 1, a total of 20 normal trichromatic observers *(*mean age 31.5 years, age range 53 years) acted as participants, whilst in experiments 2 and 3 a subset of this cohort consisting of 5 colour normal trichromats (3 males; mean age: 32 years, age range 24 years) took part. Colour vision in all normal subjects was assessed using the City University Colour Test (2nd Edition) and the HMC Anomaloscope (Oculus, Wetzlar, Germany). In experiment 4, we recorded ERGs from 3 members of a family [RM1 (31 years), RM2 (38 years) and RM3 (34 years)] and with a homozygous p.T383fsX mutation in CNGB3 causing rod monochromacy. We also recorded ERGs from 2 patients [NB1 (17 years) and NB2 (27 years)] with congenital stationary night blindness (CSNB 1) who had severely compromised rod function caused by a NYX (Xp11.4) gene mutation.

Ethical approval for this study was obtained from the local ethics committee, and all participants gave informed consent prior to the commencement of the experiments which were carried out in accord with the tenets of the Declaration of Helsinki.

## Results

### Experiment 1: Morphology of the transient rod ERG

**Fig. 2:**
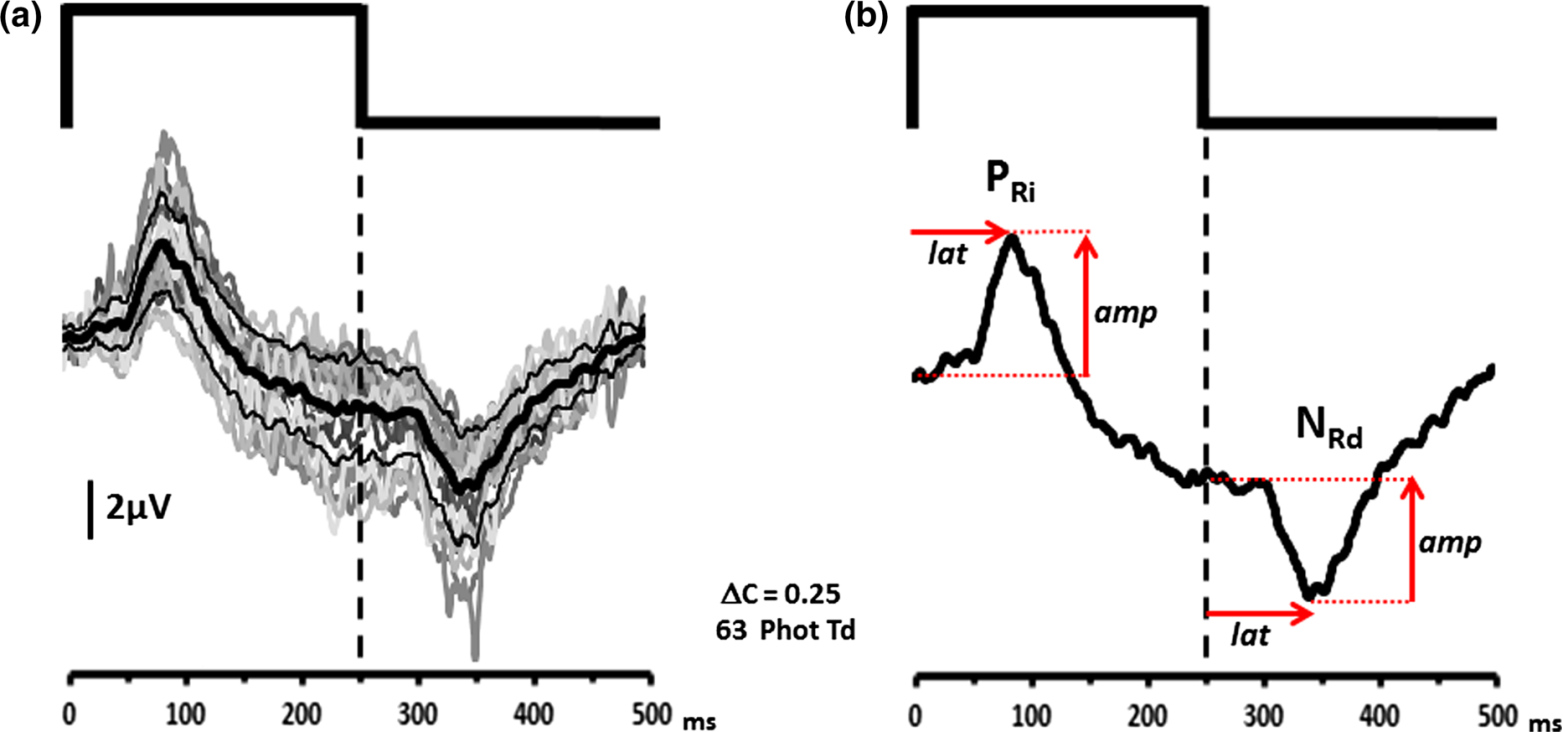
**a** Shows the individual (*grey lines*) and group averaged (*thick black line*) ERGs elicited from 20 normal participants by a silent substitution rod-isolating stimulus. The thin *black lines* represent +/− 1 S.D. from the mean. For clarity, we have shown the group averaged rod ERG in **b**, this response consists of an initial positive peak (*P*_Ri_) at stimulus onset followed by a negative response component (*N*_Rd_) after stimulus offset

Figure 2 shows ERGs obtained from 20 normal trichromatic observers in response to a silent substitution rod-isolating stimulus with a square-wave temporal profile comprising an onset (i.e. rod excitation increment) duration of 250 ms and a 250-ms offset (rod excitation decrement) period. Rod contrast, *C*_rod_ = 0.25 and the stimulus had a mean retinal illuminance of 63 ph Td. In normal trichromats, the ERG produced by this stimulus had a consistent appearance across all participants exhibiting a waveform with an initial prominent positive peak, which we have termed *P*_Ri_, which has a peak implicit time of 85.95 ms (±95% CI = 7.88 ms). The offset response is dominated by a negative component (termed *N*_Rd_) which has a mean peak implicit time of 95.18 ms (±95% CI 7.85) after the offset of the stimulus.

### Experiment 2: Rod ERGs as a function of retinal illuminance

**Fig. 3:**
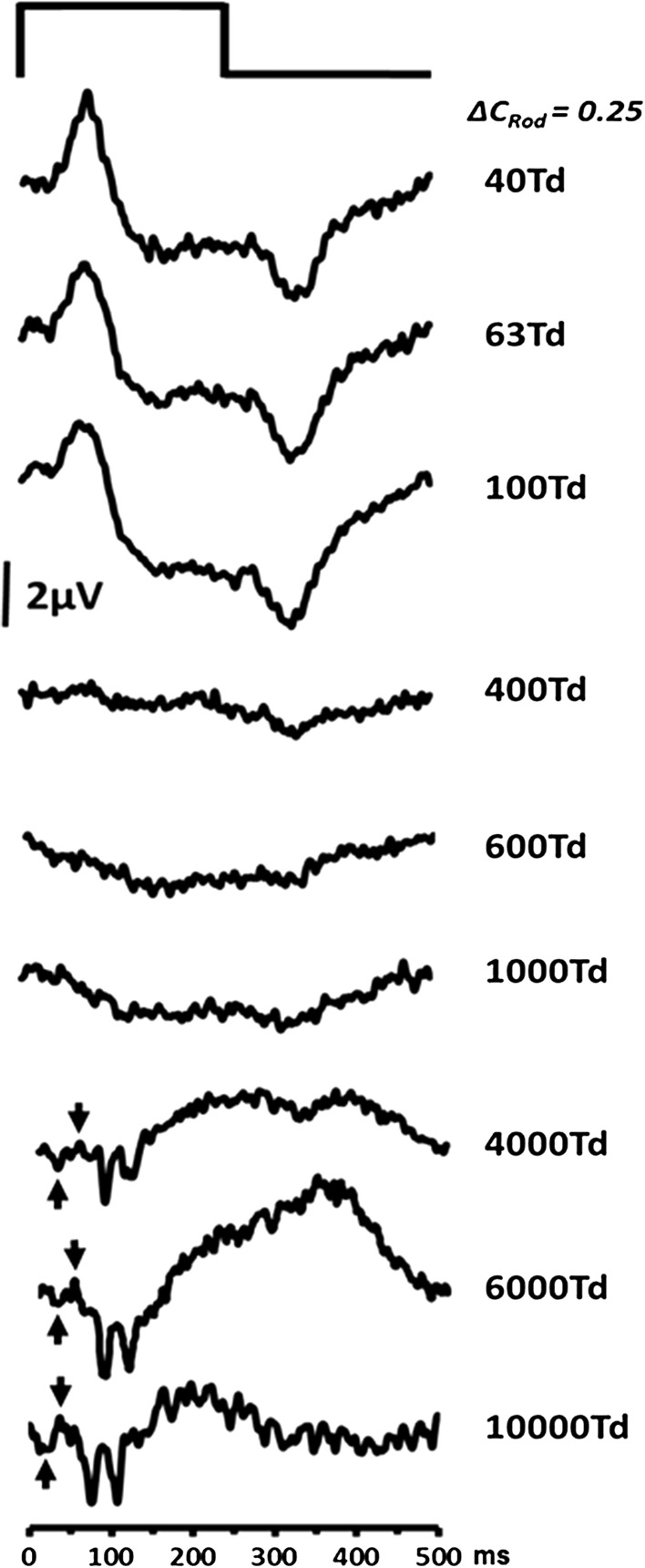
Group averaged (*n* = 5) transient rod ERG as a function of retinal illuminance. For all stimuli, the modulation of rod excitation was 0.25

ERGs mediated by rods are usually elicited from the dark-adapted eye [8] using low intensity (scotopic) stimuli [6, 8, 24–27]. However, the use of silent substitution stimuli to isolate rod activity potentially provides an opportunity to record rod responses at higher stimulus intensities. Examination of the responses elicited by stimuli that extend from mesopic to photopic levels of illumination, in particular, provide the opportunity to observe the effects of the ERG waveform as the transition from rod- to cone-mediated vision takes place. To this end, we generated a series of rod-isolating square-wave pulse stimuli which produced retinal illuminances ranging from 40 to 10,000 ph Td with a rod contrast of 0.25. Figure 3 shows the changing morphology of the averaged (*n* = 5) rod ERGs as a function of retinal illuminance. For the low intensity stimuli (40–100 ph Td), the ERGs have a distinct waveform similar to the responses shown in Fig. 2 with a prominent positive onset response (*P*_Ri_) and a negative offset (*N*_Rd_). As retinal illuminance increases from 100–1000 ph Td, the response becomes highly attenuated with hardly any discernible ERG waveform elicited by rod-isolating stimuli within this intensity range. At stimulus intensities above 1000 ph Td, a response does appear to re-emerge, but it has a very different morphology from that which is obtained at the lowest stimulus intensities. Under these conditions, the response exhibits a negative component (upward arrows in Fig. 3) with an implicit time of between 20–30 ms, followed by a small positive going peak at approximately 40 ms (downward arrows in Fig. 3). These components resemble those observed in the non-selective single flash photopic response. Later components (both positive and negative) are also observed between 75–100 ms and give the response obtained at these high illuminance levels a very different morphology to that which is observed for low illuminance levels.

### Experiment 3: ERGs elicited with non-isolating stimuli

Having examined the morphology of the ERG generated by rod-isolating silent substitution stimuli, we wanted to examine the extent to which this waveform was affected by the use of non-selective stimuli that induce excitation of cone as well as rod photoreceptors. We employed two groups of stimuli: The first were broadband flash stimuli which modulated all photoreceptors to the same extent (0.25). These stimuli were presented over a range of different retinal illuminances. The second group comprised a series of nominally rod-isolating stimuli at 63 ph Td to which varying degrees of L- and M-cone modulation were added, ranging from 0% (i.e. rod isolating) to 60% cone modulation. All stimuli had the same temporal profile as those used in experiments 1 and 2 (see Fig. 1b, c).

**Fig. 4:**
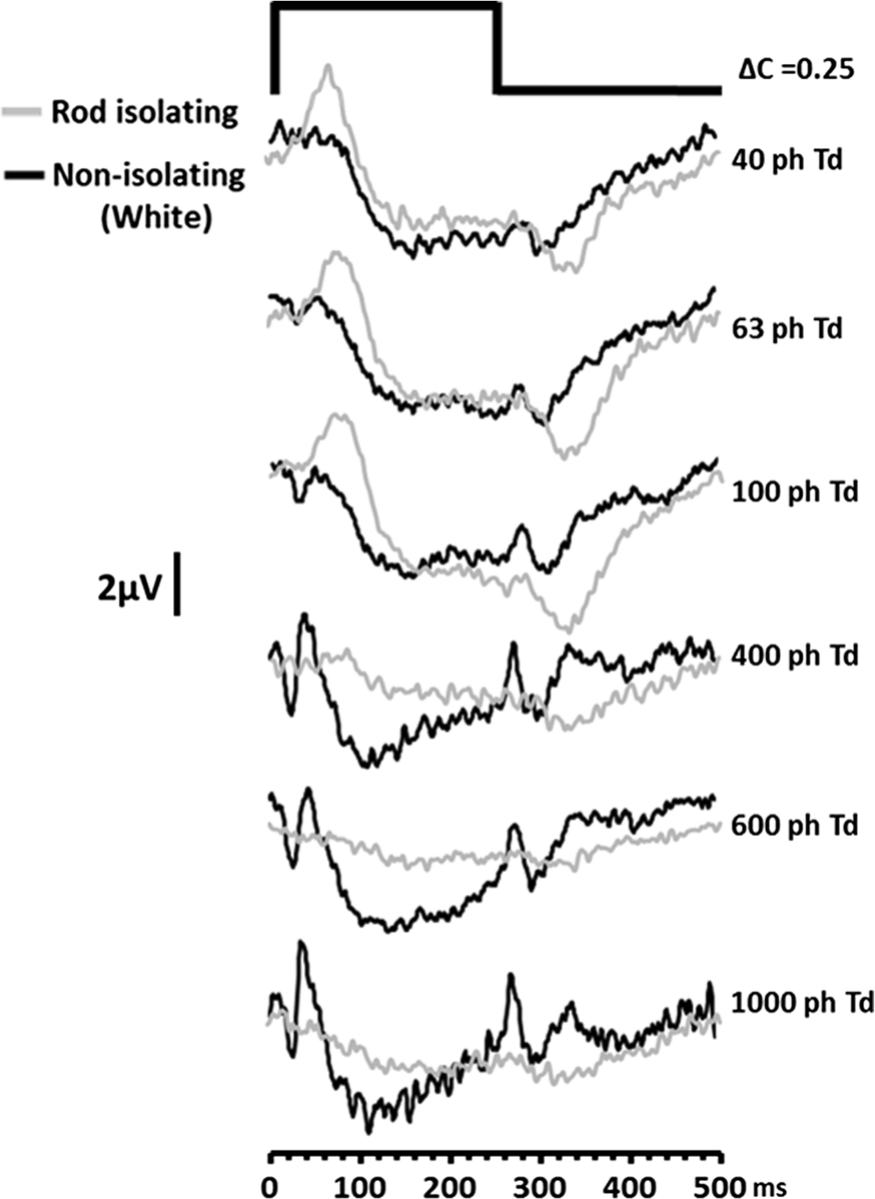
ERGs elicited by a non-isolating (*white*) stimulus of increasing intensity (*black traces*). Also shown are the responses for the rod-isolating stimuli at the same levels of retinal illuminance (*grey traces*). The traces represent group averaged (*n* = 5) responses and for all stimuli the modulation of each photoreceptor class = 0.25

Figure 4 shows the ERG responses elicited using the first non-isolating (white) group of stimuli. For comparison, the rod-isolating responses are also shown for the same stimulus intensities (grey traces). When we compare the rod-isolated responses with the non-isolated responses at similar stimulus intensities, we see that there are qualitative differences between the responses elicited by the different stimulus types. A key difference is that, at the lowest stimulus intensities, ERGs elicited by non-isolating stimuli do not exhibit the large positive component (*P*_Ri_) that is present in the rod-isolating response. Instead, non-isolated responses are dominated by a broad negativity which is similar to the scotopic threshold response (STR) that has been previously reported in the dark-adapted ERG [28, 29]. This later and longer duration negativity, also observed in the response elicited by the silent substitution stimuli at low illuminance, has previously been attributed to inner retinal activity [28], and we speculate that a similar source is responsible for the generation of this component in both the non-isolated and rod-isolated ERGs.

**Fig. 5:**
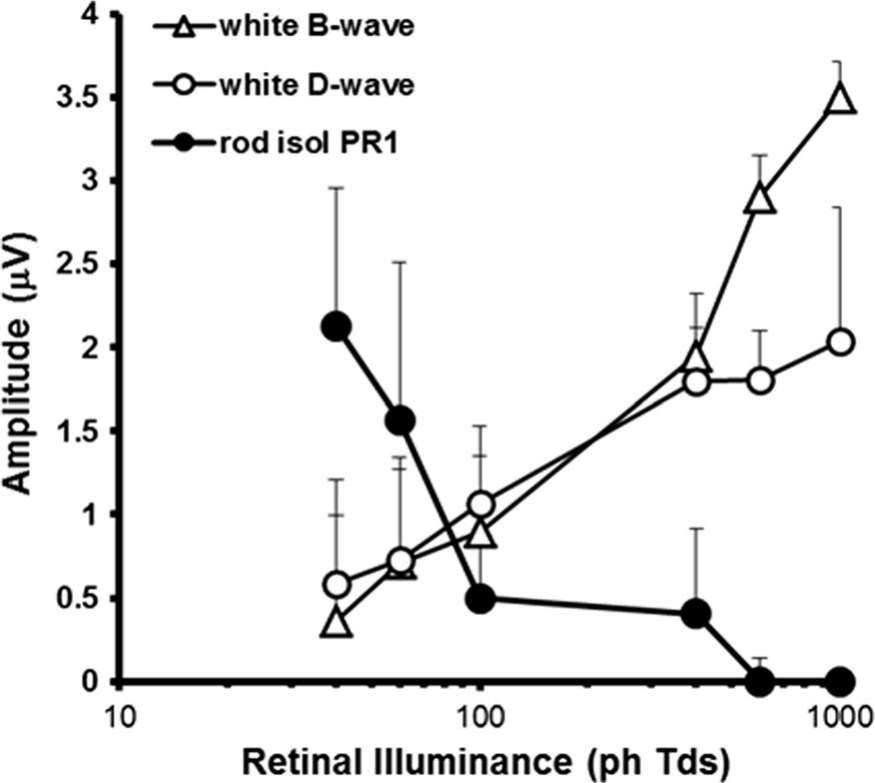
Dependency of the ERG b- (*empty triangles*) and d-wave (*empty circles*) amplitude generated by a non-selective ‘*white*’ stimulus plotted as a function of retinal illuminance. Also plotted for comparison is the amplitude of *P*_Ri_ (*filled circles*) of the rod-isolated ERG in the same participants across the same illuminance range. Data are the group averages (*n* = 5) and the *error bars* = +1 SD

As retinal illuminance increases, the non-isolated ERG starts to develop a prominent negative going a-wave and positive b-wave. Both these components have implicit times that are shorter than corresponding components found in the rod-isolated ERG. The development of these onset response components occurs in conjunction with the increased prominence of a positive d-wave offset response in the non-isolated ERG [30]. Figure 5 plots the variation in the amplitude of the b- and d-waves of the ERG generated in response to the non-isolating white stimulus as a function of retinal illuminance. As can be observed, both of these onset and offset components undergo an increase in amplitude with increasing stimulus intensity. Not unexpectedly, the waveform morphology to this non-selective stimulus takes on the appearance of the photopic on–off ERG that has been described previously (see Ref. [30], Fig. 9). In contrast, the amplitude of the *P*_Ri_ component of the rod-isolated ERG behaves very differently exhibiting a marked reduction in amplitude as a function of retinal illuminance beyond 400 ph Td.

**Fig. 6:**
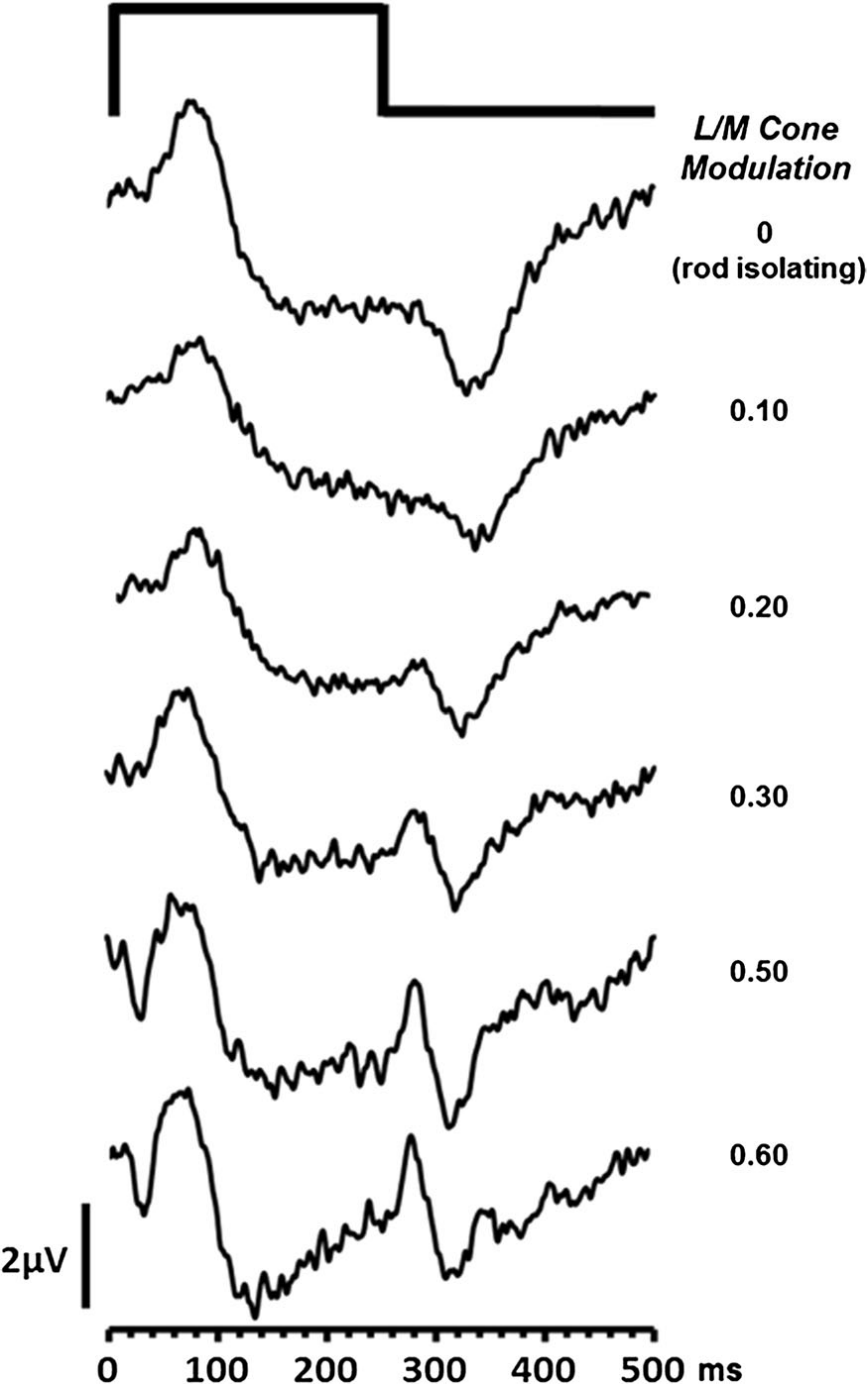
ERGs elicited by stimuli which contain increasing amounts of L- and M-cone modulation. The ERGs in the uppermost trace were generated by a stimulus that produced no L- or M-cone excitation and were therefore rod isolating. Each stimulus has a retinal illuminance = 63 photopic Trolands

**Fig. 7:**
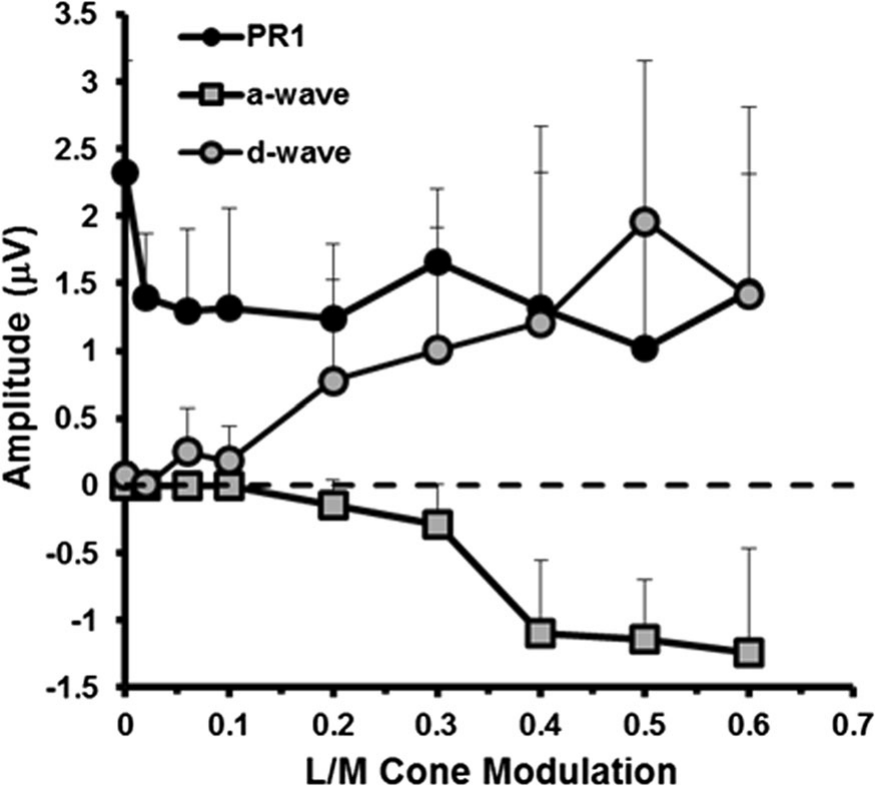
Amplitude of the a-wave (*squares*), d-wave (*circles*) and *P*_Ri_ (*black circles*) components as a function of increasing amounts of L/M-cone modulation added to a rod-isolating stimulus. Data are the group averages (*n* = 5), and the *error bars* represent +1 SD

ERGs elicited by the second group of non-isolating stimuli are shown in Fig. 6. The stimuli used in this experiment modulate L- and M-cones as well as rods. The extent of cone modulation varies across the stimuli from 0.0 (i.e. rod isolating) to 0.6. As the magnitude of cone modulation increases, there are clear changes in the ERG waveform morphology; there is an initial decrease in the *P*_Ri_ amplitude accompanied by increases in a- and d-wave amplitudes (see Fig. 7). At the highest levels of L- and M-cone modulation, the ERG waveforms elicited by these non-isolating stimuli are similar in appearance to those generated by the highest intensity white stimuli shown in Fig. 4.

### Experiment 4: Transient rod ERGs from clinical patient groups

**Fig. 8:**
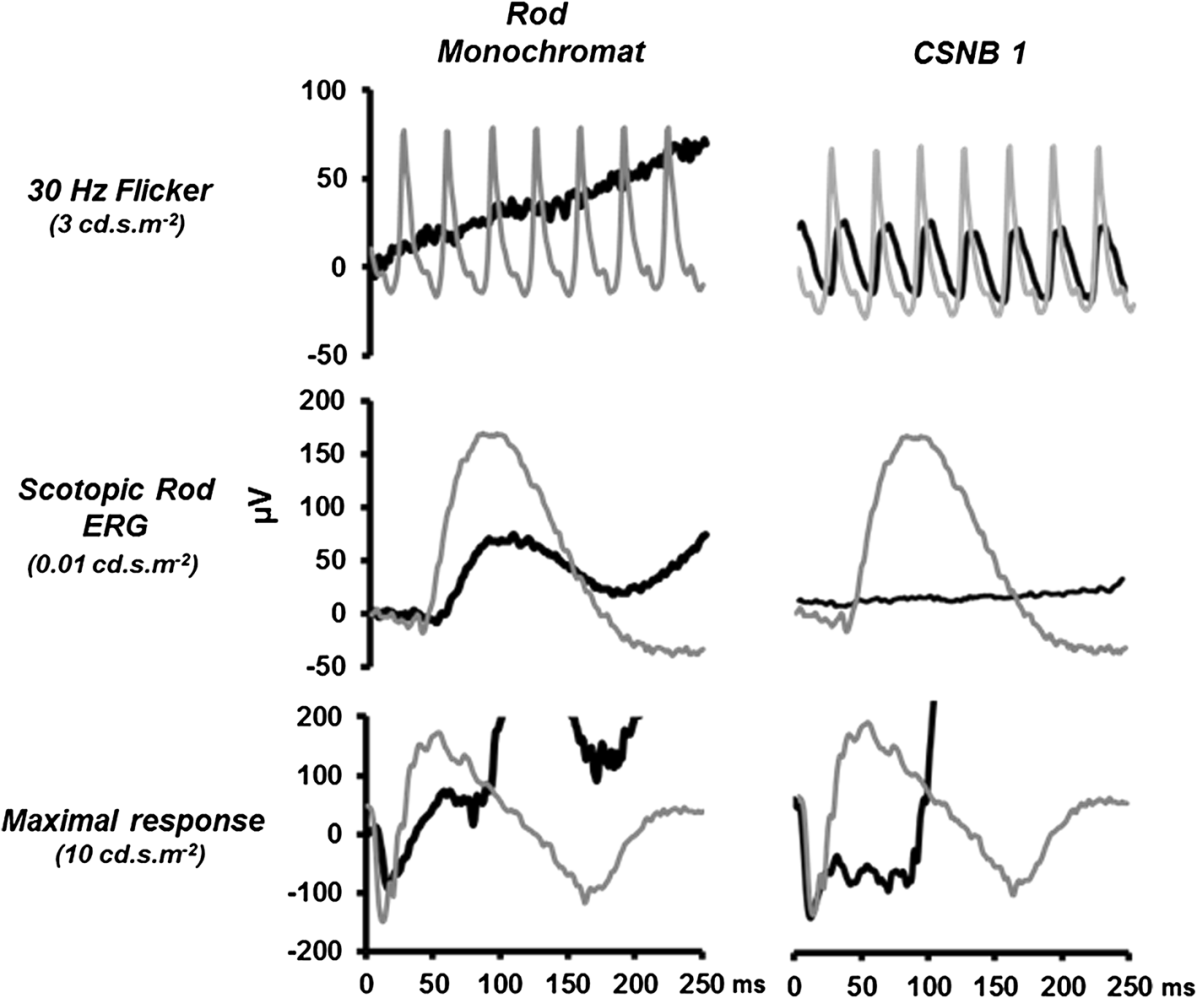
ISCEV standard 30 Hz flicker, scotopic rod and maximal response ERGs recorded from one of the rod monochromats, RM3 (*left column*) and one of the patients with CSNB1, NB1 (*right column*). The *grey traces* show the responses from a normal trichromat to these stimuli

Figure 8 shows ERGs obtained using standard ISCEV protocols [8] from one of the rod monochromats (RM3) and one of the patients with CSNB type 1 (NB1). The ERGs shown are the light-adapted 30 Hz flicker (cone), the dark-adapted scotopic (rod) and the maximal (DA10) response. Normal responses (grey traces) are also shown for comparison. As can be observed from Fig. 8, the rod monochromat has negligible cone function, as indicated by the abolished 30 Hz flicker response, but has preserved (albeit reduced) rod function [31]. In contrast, the ERGs from the CSNB subject exhibit the opposite pattern, preserved (though again reduced) responses to the 30 Hz stimulus and abolished rod function with the characteristic electronegative maximal response [18, 19, 21, 32].

**Fig. 9:**
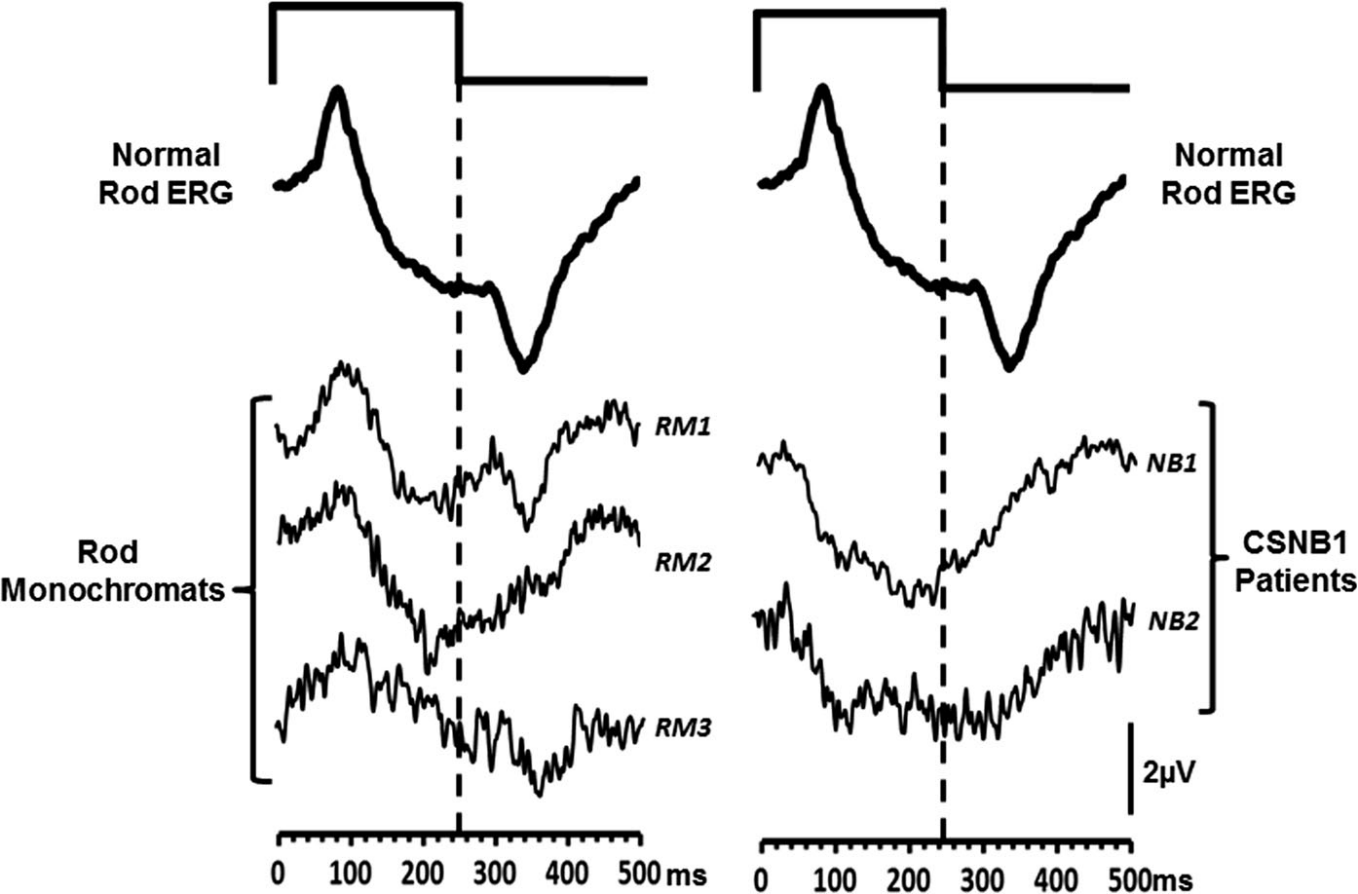
ERGs elicited from normal trichromats and from patient groups. The *left-hand column* shows ERG waveforms, elicited by a 63 ph Td rod-isolating square-wave pulse stimulus (250/250 ms onset/offset), from normal trichromats (*upper trace*). This waveform is an average of *n* = 20 observers. The *lower three traces* are the responses obtained from the three rod monochromats (RM 1-3). Traces in the *right-hand column* again show the normal rod-isolated response (*upper trace*) and ERGs obtained from two patients with CSNB 1 (NB1-2) with the same stimulus

Figure 9 shows the group averaged (*n* = 20) ERG obtained from the normal trichromats in response to the silent substitution, rod-isolating stimulus. Also shown are the responses from the three rod monochromats and 2 CSNB subjects to the same stimulus. The responses elicited from the rod monochromats exhibit similar waveform morphologies to the normal rod response, with the *P*_Ri_ and *N*_Rd_ components being identifiable at stimulus onset and offset, respectively. However, response amplitudes vary across the three patients, and there is inter-subject variation in terms of the quality of waveform appearance. This is largely due to the fact that rod function is compromised in all of these individuals. The canonical view of rod monochromacy is that it primarily leads to cone dysfunction, leaving rod function intact (see Ref. [33]). However, Fig. 8 clearly demonstrates an attenuated ISCEV scotopic rod response for subject RM3 (the rod monochromat with the largest deficit in the rod response), and this is also the case for subjects RM1 and RM2 (data not shown), the latter subject being the least affected out of the three in terms of rod dysfunction. This secondary loss of rod response in rod monochromats is consistent with reports from previous studies [17, 34, 35].

In contrast, the ERGs generated by the rod-isolating stimuli from the CSNB patients are markedly different. The responses lack a prominent *P*_Ri_ component; instead, the waveform elicited by contrast increment (onset) is dominated by a prolonged negative component. The offset response is also very different in that it shows a small positivity rather than a large negativity.

## Discussion

In this study, we have used silent substitution stimuli to elicit transient ERGs from the light-adapted human retina in an attempt to generate retinal responses that selectively reflect rod-mediated visual function. We have characterised the morphology of this rod ERG waveform in the normal trichromatic retina and demonstrated how non-selective stimuli induce changes in this response that arise as the result of cone photoreceptor stimulation. Importantly, we have shown that rod ERGs generated by our methodology exhibit a clear reduction in response amplitude as stimulus intensity increases from mesopic to photopic levels. This response attenuation is not observed in ERGs elicited by stimuli that are not rod selective and is critical because it provides a clear correlation with rod photoreceptor response properties which exhibit response saturation over the same illumination range [36]. Complementing our observations from the normal human retina are the responses from participants with two contrasting kinds of inherited retinal pathology that have either selectively preserved (rod monochromacy), or compromised (CSNB) rod function. The similarity between the waveform morphologies of ERGs obtained by rod-isolating stimuli from normal trichromats and those from rod monochromats provides further verification that silent substitution stimuli can effectively isolate rod-mediated activity in the light-adapted trichromatic retina. Furthermore, the fact that key features of our ‘normal’ rod ERG waveform are absent in CSNB subjects who have compromised rod function, but preserved cone function, provides another indicator that this methodology does provide a selective assay of rod photoreceptor function.

### Origins of on and off components in the Rod ERG

The human dark-adapted rod ERG, recorded under ISCEV standard conditions [8], typically comprises a positive b-wave of large amplitude with an implicit time of approximately 100 ms (see Fig. 8). The response is generated by a short duration broadband flash stimulus, and the resultant waveform is in effect a composite response of both onset and offset components (though heavily dominated by the former). In the mammalian retina, low scotopic vision is mediated by a pathway based upon rods which synapse with depolarising rod bipolar cells [30, 37–39] and numerous pharmacological studies point to the direct involvement of this pathway in the generation of the dark-adapted ERG b-wave [40–42]. ERGs elicited from the normal light-adapted human retina to the onset of a rod-isolating silent substitution stimulus (<400 ph Td) also are dominated by an initial positive component, *P*_Ri_, with an implicit time of 85.95 ms (±95% CI 7.88 ms). We propose that the origin of this component is similar to that of the dark-adapted rod b-wave or the PII response [28, 43, 44] and is produced by the depolarisation of the rod ON-bipolar cells [28, 30]. Our recordings from participants with type 1 CSNB provide support for this view. This form of CSNB is the direct result of ON-bipolar cell dysfunction, and individuals with this condition have a characteristic set of full-field ERG abnormalities, abolished scotopic responses, electronegative scotopic bright flash ERGs as well as abnormalities in the morphology of the photopic a-wave [18–21]. The ERGs we have recorded from these individuals using the rod-isolating silent substitution stimuli lack any obvious *P*_Ri_ component but, in keeping with previous findings [e.g. 30], exhibit an electronegative waveform in response to the onset of a long duration rod-isolating stimuli. The rod ERGs obtained from the CSNB participants contrast with those elicited from rod monochromats and normal trichromats. The rod monochromats are members of a family with a homozygous pT383fsX mutation in the CNGB3 gene. This mutation generates deficits in critical parts of cone phototransduction cycle and leads to a loss of cone function. Individuals with this condition typically present with photophobia, nystagmus, reduced visual acuity and a total loss of colour vision but have preserved rod function [17, 31, 45, 46]. The fact that the silent substitution rod-isolating stimulus generates an ERG from these individuals that has the same basic morphology as the rod ERG obtained from the normal retina provides verification that this response does indeed reflect rod-mediated retinal function. This is despite the fact that light-adapted trichromatic retina also contains functional cone as well as rod photoreceptors.

The temporally extended nature of our stimulus means that an offset response is also a feature of our rod ERG responses—something that is not usually observed in the ISCEV scotopic ERG. An intense, long duration stimulus typically evokes a positive potential or d-wave from cone-rich light-adapted retinas at stimulus offset [44, 47]. Examples of this offset response component can be seen in the ERGs recorded in response to high intensity white stimuli and stimuli which induce cone and rod excitation (see Figs. 4, 6). In comparison, offset responses elicited from dark-adapted, rod-dominated retinas comprise a negative component followed by a slower positive response [43, 44]. These morphological features are more in keeping with those observed in our rod-isolated ERGs which at stimulus offset exhibit a negative trough, *N*_Rd_, that typically occurs at 95 ms post stimulus offset. The rod ERG offset response was first described when assessing retinal responses to long duration stimuli in rod dominant animal models and was described as a corneal negative wave occurring after stimulus offset [43, 48]. Brown originally suggested the offset response was a combination of the decay of ON-bipolar cells plus a dc component along with the recovery of the photoreceptors [42]. Further analysis in the cat confirmed that part of the negative trough is formed by repolarisation of the rod bipolar cells but that the slow positivity, immediately following it, originates in the more proximal regions of the retina [42]. The literature on the rod offset response in human retina is limited [49, 50]. In one study [49], the rod offset ERG was recorded in a patient with S-cone monochromacy using silent substitution. The resultant response is qualitatively similar to the offset ERGs reported in this study. A second study [50] used scotopic rapid on/off ramp stimuli and multifocal stimuli to record the rod onset and offset responses. The elicited waveform had a positive deflection at onset and a negative dip at offset. Our speculation is that the negative offset component observed in the rod-isolated ERGs recorded in this study is related to the recovery of the ON-bipolars, rather than an independent entity. The fact that a negative offset component is not observed in ERGs recorded from CSNB patients may provide further support for this notion. In these patients, the ON-bipolars are dysfunctional, and there is a lack of response at stimulus onset. As a consequence, there is no recovery following stimulus offset.

### Rod ERGs as a function of retinal illuminance

A key feature of the ERGs generated by the silent substitution rod-isolating stimuli is that they exhibit a decrease in amplitude with increasing stimulus intensity, the responses becoming highly attenuated above 100 ph Td. This decrease is significant because it occurs across the range of mesopic illumination levels for which the saturation of rod responses is purported to begin [32, 51, 52]. This intensity-dependent decrease in amplitude for the isolate rod ERG is in stark contrast to the increase in amplitude of the responses elicited by non-selective stimuli which not only modulate rods but also cone photoreceptors (Figs. 3, 4, 5). This response behaviour provides another piece of evidence which points to the selective isolation of rod function by the current stimulation protocols. Similar intensity-dependent increases and decreases have been demonstrated in the mouse retina for cone and rod-mediated ERGs, respectively [14]. Interestingly, similar to the murine responses, human rod ERGs appear to undergo a similar abrupt reduction in amplitude across a relatively narrow range of retinal illuminance. The rapid nature of the rod response attenuation, which is coupled with an increase in the ERGs generated by cone photoreceptors [14], has prompted speculation about the existence of retinal mechanisms which control the switch from rod- to cone-mediated vision with increasing retinal illumination. One possibility is that rod response levels are moderated by the light intensity experienced by cones [53, 54]. Various mechanisms have been proposed as to how this suppression of rod function might be achieved, including mediation via gap junctions that exist between rods and cones [55] or via neural switching mechanisms involved cone bipolars [56]. These have, thus far, only been described in the mouse retina—but the behaviour of the rod-isolated ERGs shown here, suggesting that similar mechanisms involving the rapid suppression of rod responses by increasing cone activity exist in the human retina. The use of rod-isolating silent substitution stimuli may provide a means via which these mechanisms can be studied in humans.

Our results show that whilst there is a clear attenuation of the rod-isolated ERG for stimuli above 100 ph Td, some form of response does re-emerge at high stimulus illuminances (≥4000 ph Td). However, the morphology of these waveforms is clearly very different from that obtained using low illuminance stimuli (see Fig. 3). The early negative and positive components, occurring at approximately 20 and 40 ms, respectively, are similar in timing to the a- and b-waves observed in ERGs generated by non-selective stimuli. In addition, there is a later complex of negative and positive components, occurring between 75–110 ms that is observed in the high illuminance responses. This complex is completely absent from the responses elicited by the optimal (<100 ph Td) rod-isolating stimuli. In the light of these differences in waveform morphology, our view is that the ERGs elicited by rod-isolating stimuli of high illuminance no longer selectively reflect rod function and are the result of contamination from non-rod-mediated sources. Previous work has demonstrated that cone photoreceptors may form one potential source of these intrusions. This is based on the fact that the temporal response limit of these high illuminance ERGs far exceeds that supportable by the rod system and lies closer to temporal response limit of the cones [13]. These intrusions may be the result of the intrinsic anatomical connectivity that exists between the rod signalling pathway and cones [34, 57, 58]. The inadvertent stimulation of other photoreceptor populations may also arise as a result of departures in the degree rod isolation provided by our stimuli. Silent substitution calculations are based on representative photoreceptor fundamentals [22]. However, across individuals there are differences in these fundamentals, as well as variation in pre-retinal absorption characteristics. These factors are likely to increase the likelihood of stimulation of other photoreceptor classes which becomes more significant with increasing stimulus intensity. In addition to retinal-based sources of contamination, we also cannot rule out the possibility of myogenic contamination (due to blinks or blepharospasm) that is often induced by stimuli of high intensities. This could form a potential source, particularly for the later components observed in the ERGs elicited by high illuminance stimuli. Our results suggest that even with silent substitution stimuli, which in theory should elicit no cone excitation, rod isolation can no longer be assured for stimuli of illuminance above 1000 ph Td as a result of these potential physiological and physical sources of contamination.

In summary, we have described the key features of an ERG response, generated by silent substitution stimulation, which selectively reflect the operation of rod photoreceptors in the normal, light-adapted human retina. We have demonstrated how this rod ERG is affected by the use of stimuli that vary in the extent to which they selectively isolate rod function. In addition, we have also shown how this response is influenced by retinal pathologies that differentially affect rod and cone function in humans. We propose that our methodology will prove to be useful in the respect that it provides an opportunity for the examination of human rod function, in both the normal and abnormal retina, without having to subject participants to long periods of dark adaptation. Secondly, the use of rod-isolating stimuli, used in conjunction with carefully generated stimuli that are less selective in terms of their rod isolation, provides a means to study interactions between rods and cones in the normal and pathological retina, particularly in the context of the control of retinal sensitivity across mesopic illumination levels.
